# Identification of genetic associations and functional SNPs of bovine *KLF6* gene on milk production traits in Chinese holstein

**DOI:** 10.1186/s12863-023-01175-w

**Published:** 2023-11-28

**Authors:** Yanan Liu, Bo Han, Weijie Zheng, Peng Peng, Chendong Yang, Guie Jiang, Yabin Ma, Jianming Li, Junqing Ni, Dongxiao Sun

**Affiliations:** 1 Key Laboratory of Animal Genetics, Breeding and Reproduction of Ministry of Agriculture and Rural Affairs, National Engineering Laboratory for Animal Breeding, Department of Animal Genetics, Breeding and Reproduction, College of Animal Science and Technology, China Agricultural University, No. 2 Yuanmingyuan West Road, Haidian District, Beijing, 100193 China; 2 Hebei Province Animal Husbandry and Fine Breeds Work Station, No. 7 Xuefu Road, Changan District, Shijiazhuang, 050000 China

**Keywords:** *KLF6*, SNP, Genetic effect, Milk production traits, Chinese Holstein

## Abstract

**Background:**

Our previous research identified the *Kruppel like factor 6* (*KLF6*) gene as a prospective candidate for milk production traits in dairy cattle. The expression of *KLF6* in the livers of Holstein cows during the peak of lactation was significantly higher than that during the dry and early lactation periods. Notably, it plays an essential role in activating peroxisome proliferator-activated receptor α (PPARα) signaling pathways. The primary aim of this study was to further substantiate whether the *KLF6* gene has significant genetic effects on milk traits in dairy cattle.

**Results:**

Through direct sequencing of PCR products with pooled DNA, we totally identified 12 single nucleotide polymorphisms (SNPs) within the *KLF6* gene. The set of SNPs encompasses 7 located in 5′ flanking region, 2 located in exon 2 and 3 located in 3′ untranslated region (UTR). Of these, the g.44601035G > A is a missense mutation that resulting in the replacement of arginine (CGG) with glutamine (CAG), consequently leading to alterations in the secondary structure of the KLF6 protein, as predicted by SOPMA. The remaining 7 regulatory SNPs significantly impacted the transcriptional activity of *KLF6* following mutation (*P* < 0.005), manifesting as changes in transcription factor binding sites. Additionally, 4 SNPs located in both the UTR and exons were predicted to influence the secondary structure of *KLF6* mRNA using the RNAfold web server. Furthermore, we performed the genotype-phenotype association analysis using SAS 9.2 which found all the 12 SNPs were significantly correlated to milk yield, fat yield, fat percentage, protein yield and protein percentage within both the first and second lactations (*P* < 0.0001 ~ 0.0441). Also, with Haploview 4.2 software, we found the 12 SNPs linked closely and formed a haplotype block, which was strongly associated with five milk traits (*P* < 0.0001 ~ 0.0203).

**Conclusions:**

In summary, our study represented the *KLF6* gene has significant impacts on milk yield and composition traits in dairy cattle. Among the identified SNPs, 7 were implicated in modulating milk traits by impacting transcriptional activity, 4 by altering mRNA secondary structure, and 1 by affecting the protein secondary structure of *KLF6*. These findings provided valuable molecular insights for genomic selection program of dairy cattle.

**Supplementary Information:**

The online version contains supplementary material available at 10.1186/s12863-023-01175-w.

## Background

In recent years, researchers have implemented multiple strategies, such as genome-wide association study (GWAS) [1–5], RNA sequencing (RNA-seq) [6–9], whole genome bisulfite sequencing (WGBS) [10–14], signature selection [15–18], to explore the genetic mechanisms underlying complex traits in human, mouse and domestic animals, and numerous functional genes and genetic associations have been identified. In our previous RNA-seq study, we found a significant upregulation of the *Kruppel-like factor 6* (*KLF6*) gene in the livers of Holstein cows during the peak lactation period in comparison to both the dry and early lactation periods [19]. The KLF6 protein participates in lipid metabolic processes by activating peroxisome proliferator-activated receptor α (PPARα) signaling pathway. Notably, *KLF6* was located near the peak regions of the known quantitative trait loci (QTL) for milk fat yield (0.09 Mb), milk fat percentage (0.28 Mb), milk protein yield (0.45 Mb) and protein percentage (1.38 Mb) (https://www.animalgenome.org/cgi-bin/QTLdb/index). Therefore, we considered the *KLF6* gene as a promising candidate for milk production traits.

The *KLF6* gene is located on bovine chromosome 13 and consists of 2 exons, with a total length of 8460 base pairs (bp). The KLF6 protein belongs to the specificity proteins/Krüppel-like factor (SP/KLF) transcription factors family, known for its regulation of various genes involved in fundamental cellular and biological processes. These processes encompass vascular remodeling [20], cell differentiation, proliferation, cycle and apoptosis [21–23], tissue development and growth [24–27] in humans.

Overexpression of *KLF6* has been demonstrated to increase the number of lipid droplets and elevate the expression of peroxisome proliferator-activated receptor-γ (PPARγ) and CCAAT/enhancer binding protein α (C/EBPα) in pig adipose-derived stem cells. Conversely, treatment with small interfering RNAs (siRNAs) targeting *KLF6* resulted in the opposite consequence [28]. The knocked-down of *KLF6* negatively influences both adipogenesis and differentiation in primary adipocyte cells [29]. Moreover, polymorphisms associated with the KLF6 gene have shown significant correlations with intramuscular fat traits in Qinchuan cattle [30]. Nevertheless, no prior investigations have explored the influence of the *KLF6* gene on milk traits in dairy cattle. Thus, the primary objective of the present study was to ascertain the genetic effects of the *KLF6* gene on milk yield and composition traits and to identify potential functional genetic variations in Chinese Holstein cattle population.

## Results

### SNPs identification

**Fig. 1:**
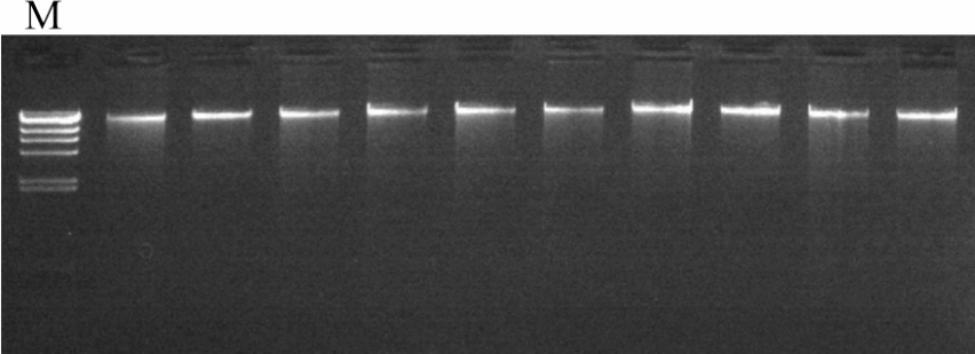
Agarose gel electrophoresis result of 10 DNA samples extracted from the blood samples of cows. Lane 1 is λDNA /HindIII Marker

We extracted genomic DNA from 1123 blood samples of Chinese Holstein cows (Fig. 1). By sequencing the PCR products of the *KLF6* gene for the entire coding sequence and 2000 bp of the 5’ and 3’ flanking regions, we identified a total of 12 SNPs, including seven in 5′ flanking region, two in exon 2 and three in 3′ UTR. Among these, the g.44601035G > A was a missense mutation that changed from arginine to glutamine of the amino acid. The genotype and allele frequencies of the identified SNPs were presented in Table 1.

**Table 1:** Detailed information of 12 SNPs identified in the *KLF6* gene

SNP	Location	GenBank accession No.	Genotype (No.)	Allele	Allelic Frequency
g.44,595,327 A > G	5′ flanking region	rs41692335	AA (165)	A	0.3894
			AG (540)	G	0.6106
			GG (412)		
g.44595580G > A	5′ flanking region	rs110464810	AA (412)	A	0.6115
			AG (542)	G	0.3885
			GG (163)		
g.44595695G > A	5′ flanking region	rs110289079	AA (411)	A	0.6096
			AG (541)	G	0.3904
			GG (166)		
g.44,595,808 C > T	5′ flanking region	rs208019372	CC (615)	C	0.7449
			CT (434)	T	0.2551
			TT (68)		
g.44,596,221 A > C	5′ flanking region	rs211266130	AA (614)	A	0.7457
			AC (432)	C	0.2543
			CC (67)		
g.44,596,271 C > T	5′ flanking region	rs29024529	CC (526)	C	0.6889
			CT (487)	T	0.3111
			TT (104)		
g.44,596,874 A > G	5′ flanking region	rs41692337	AA (169)	A	0.3885
			AG (502)	G	0.6115
			GG (410)		
g.44,600,440 C > T	Exon-2	rs209109676	CC (653)	C	0.7675
			CT (407)	T	0.2325
			TT (56)		
g.44601035G > A	Exon-2	rs211273884	AA (58)	A	0.2297
			AG (394)	G	0.7703
			GG (658)		
g.44,601,887 C > T	3’ UTR	rs41692344	CC (468)	C	0.6464
			CT (521)	T	0.3536
			TT (138)		
g.44602809G > T	3’ UTR	rs208700974	GG (856)	G	0.8790
			GT (250)	T	0.1210
			TT (10)		
g.44603687G > A	3’ UTR	rs41692345	AA (419)	A	0.6145
			AG (536)	G	0.3855
			GG (163)		

### Associations between SNPs and the five milk production traits

Using SAS 9.2, we assessed the genetic associations between the 12 identified SNPs and five milk traits in Holstein cows, including milk yield, fat yield, protein yield, fat percentage and protein percentage (Table 2).

**Table 2:** Associations of the SNPs in *KLF6* gene with milk production traits in two lactations in Chinese Holstein (LSM ± SE).

SNP	Lactation	Genotype (No.)	Milk yield, kg	Fat yield, kg	Fat percentage, %	Protein yield, kg	Protein percentage, %
g.44,595,327 A > G	1	AA(165)	6928.35 ± 255.38	263.82 ± 10.364^a^	3.799 ± 0.102^a^	219.8 ± 5.025^a^	3.165 ± 0.037^a^
		AG(540)	6915.51 ± 254.56	259.4 ± 10.34^a^	3.736 ± 0.102^b^	214.69 ± 5.027^b^	3.125 ± 0.037^b^
		GG(412)	6808.59 ± 254.37	254.3 ± 10.333^b^	3.722 ± 0.102^b^	212.6 ± 5.025^b^	3.131 ± 0.037^b^
		*P* value	0.0509	0.0009**	0.0155*	< 0.0001**	0.0001**
	2	AA(107)	9686.93 ± 142.82^a^	372.34 ± 5.951^a^	3.833 ± 0.058^a^	303.62 ± 4.339^a^	3.147 ± 0.02
		AG(307)	9442.31 ± 129.56^b^	352.4 ± 5.462^b^	3.758 ± 0.053^b^	293.01 ± 3.981^b^	3.129 ± 0.018
		GG(200)	9460.75 ± 132.74^b^	349.51 ± 5.568^b^	3.696 ± 0.054^c^	296.97 ± 4.059^b^	3.151 ± 0.018
		*P* value	0.0052**	< 0.0001**	0.0001**	< 0.0001**	0.04*
g.44595580G > A	1	AA(412)	6629.68 ± 250.39	249.98 ± 10.157^b^	3.775 ± 0.1^b^	203.9 ± 4.905^b^	3.119 ± 0.036^a^
		AG(542)	6722.68 ± 250.81	255.81 ± 10.176^a^	3.806 ± 0.1	204.96 ± 4.918^b^	3.117 ± 0.036^a^
		GG(163)	6754.22 ± 252.28	260.12 ± 10.23^a^	3.861 ± 0.101^a^	209.69 ± 4.937^a^	3.155 ± 0.036^b^
		*P* value	0.074	0.0002**	0.0055**	< 0.0001**	0.0002**
	2	AA(200)	9566.8 ± 133.86^b^	344.42 ± 5.611^b^	3.616 ± 0.054^b^	304.7 ± 4.09^b^	3.177 ± 0.019
		AG(307)	9614.5 ± 130.15^b^	349.3 ± 5.488^b^	3.67 ± 0.053^b^	303.73 ± 4^b^	3.162 ± 0.018
		GG(107)	9831.6 ± 141.34^a^	368.39 ± 5.895^a^	3.75 ± 0.057^a^	313.34 ± 4.298^a^	3.181 ± 0.02
		*P* value	0.0042**	< 0.0001**	0.0002**	< 0.0001**	0.1567
g.44595695G > A	1	AA(411)	6830.13 ± 249.78	247.71 ± 10.116^b^	3.662 ± 0.1	208.46 ± 4.868^b^	3.116 ± 0.036^b^
		AG(541)	6906.41 ± 250.41	254.04 ± 10.14^a^	3.702 ± 0.1	210.18 ± 4.877	3.117 ± 0.036^b^
		GG(166)	6898.9 ± 251.68	253.42 ± 10.194	3.722 ± 0.101	212.35 ± 4.911^a^	3.155 ± 0.036^a^
		*P* value	0.252	0.0029**	0.0335*	0.0075**	< 0.0001**
	2	AA(199)	9471.29 ± 132.89^b^	349.54 ± 5.575^b^	3.692 ± 0.054^b^	297.44 ± 4.064^b^	3.154 ± 0.018^a^
		AG(307)	9432.68 ± 129.56^b^	351.85 ± 5.461^b^	3.757 ± 0.053^b^	292.56 ± 3.981^c^	3.127 ± 0.018^b^
		GG(108)	9678.43 ± 142.38^a^	372.25 ± 5.933^a^	3.835 ± 0.058^a^	303.48 ± 4.326^a^	3.149 ± 0.02
		*P* value	0.0059**	< 0.0001**	< 0.0001**	< 0.0001**	0.0096**
g.44,595,808 C > T	1	CC(615)	6951.08 ± 250	258.45 ± 10.158	3.723 ± 0.1	214.74 ± 4.94^b^	3.117 ± 0.036
		CT(434)	6912.47 ± 251.39	257.52 ± 10.217	3.73 ± 0.101	213.44 ± 4.975^b^	3.109 ± 0.036^b^
		TT(68)	7008.87 ± 261.67	260.58 ± 10.617	3.709 ± 0.105	222.01 ± 5.146^a^	3.144 ± 0.038^a^
		*P* value	0.4903	0.6825	0.8303	< 0.0001**	0.0341*
	2	CC(357)	9501.42 ± 129.08^a^	353.3 ± 5.443	3.725 ± 0.052	296.64 ± 3.967^a^	3.14 ± 0.018
		CT(223)	9466.48 ± 132.05^a^	354.22 ± 5.547	3.766 ± 0.054	294.86 ± 4.044^a^	3.137 ± 0.018
		TT(34)	9127.73 ± 171.65^b^	345.14 ± 7.009	3.804 ± 0.069	284.4 ± 5.112^b^	3.12 ± 0.024
		*P* value	0.0096**	0.1787	0.0854	0.0021**	0.5218
g.44,596,221 A > C	1	AA(614)	6643.76 ± 252.53	263.94 ± 10.247	3.943 ± 0.101	203.2 ± 4.991^a^	3.123 ± 0.036
		AC(432)	6547.53 ± 254.24	260.41 ± 10.324	3.948 ± 0.102	200.21 ± 5.044^b^	3.12 ± 0.037
		CC(67)	6702.38 ± 264.48	268.41 ± 10.725	3.963 ± 0.106	206.73 ± 5.217^a^	3.14 ± 0.038
		*P* value	0.056	0.0383*	0.8526	< 0.0001**	0.3585
	2	AA(355)	9551.93 ± 130.72^a^	352.35 ± 5.517	3.677 ± 0.053	308.12 ± 4.021^a^	3.22 ± 0.018
		AC(222)	9464.89 ± 134.17^a^	351.19 ± 5.642	3.717 ± 0.054	303.82 ± 4.112^b^	3.209 ± 0.019
		CC(32)	9150.13 ± 177.15^b^	344.95 ± 7.229	3.774 ± 0.071	295.13 ± 5.272^b^	3.2 ± 0.025
		*P* value	0.0041**	0.312	0.0557	0.0002**	0.2886
g.44,596,271 C > T	1	CC(526)	6788.7 ± 249.29^b^	250.62 ± 10.096^a^	3.727 ± 0.1^a^	208.53 ± 4.86^b^	3.139 ± 0.036^a^
		CT(487)	6983.21 ± 249.35^a^	253.75 ± 10.101^a^	3.665 ± 0.1^b^	213.59 ± 4.864^a^	3.123 ± 0.036^b^
		TT(104)	6706.52 ± 257.3^b^	242.32 ± 10.412^b^	3.664 ± 0.103	202.46 ± 5.001^c^	3.104 ± 0.037^b^
		*P* value	< 0.0001**	0.0008**	0.0019**	< 0.0001**	0.0022**
	2	CC(304)	9397.7 ± 130.62^b^	364.89 ± 5.5^a^	3.917 ± 0.053^a^	299.72 ± 4.009^b^	3.194 ± 0.018
		CT(258)	9372.63 ± 131.9^b^	349.18 ± 5.549^b^	3.769 ± 0.054^b^	298.57 ± 4.045^b^	3.194 ± 0.018
		TT(51)	9722.82 ± 157.38^a^	354.6 ± 6.482^b^	3.64 ± 0.063^c^	314.57 ± 4.727^a^	3.227 ± 0.022
		*P* value	0.0032**	< 0.0001**	< 0.0001**	< 0.0001**	0.0841
g.44,596,874 A > G	1	AA(169)	6621.56 ± 260.53	251.31 ± 10.558	3.796 ± 0.104	208.09 ± 5.123^a^	3.157 ± 0.038^a^
		AG(502)	6742.08 ± 259.51^a^	256.1 ± 10.52^a^	3.797 ± 0.104	207.99 ± 5.112^a^	3.125 ± 0.038^b^
		GG(410)	6621.1 ± 259.41^b^	248.33 ± 10.521^b^	3.754 ± 0.104	204.89 ± 5.118^b^	3.125 ± 0.037^b^
		*P* value	0.0227*	0.0003**	0.0618	0.0014**	0.0016**
	2	AA(105)	9626.84 ± 144.15^a^	349.38 ± 6.022^a^	3.67 ± 0.058^a^	309.84 ± 4.39^a^	3.216 ± 0.02
		AG(287)	9363.44 ± 134.21^b^	330.33 ± 5.656^b^	3.603 ± 0.054^a^	298.9 ± 4.123^b^	3.202 ± 0.018^b^
		GG(198)	9424.69 ± 138.2^b^	327.25 ± 5.804^b^	3.524 ± 0.056^b^	304.46 ± 4.231^a^	3.227 ± 0.019^a^
		*P* value	0.0039**	< 0.0001**	< 0.0001**	< 0.0001**	0.0339*
g.44,600,440 C > T	1	CC(653)	6990.22 ± 250.02^a^	256.28 ± 10.152^a^	3.686 ± 0.1^a^	216.18 ± 4.927^a^	3.116 ± 0.036
		CT(407)	7065.68 ± 250.65^a^	256.04 ± 10.18^a^	3.64 ± 0.1^b^	217.15 ± 4.949^a^	3.105 ± 0.036
		TT(56)	6575.48 ± 265.06^b^	245.03 ± 10.757^b^	3.72 ± 0.106	202.51 ± 5.23^b^	3.112 ± 0.038
		*P* value	< 0.0001**	0.0189*	0.017*	< 0.0001**	0.2944
	2	CC(368)	9467.17 ± 129.76^a^	364.43 ± 5.474^a^	3.888 ± 0.053^a^	300.43 ± 3.99^b^	3.182 ± 0.018^c^
		CT(215)	9266.39 ± 133.32^b^	347.15 ± 5.594^b^	3.775 ± 0.054^b^	297.32 ± 4.078^b^	3.211 ± 0.018^b^
		TT(30)	9711.85 ± 174.73^a^	353.83 ± 7.13	3.656 ± 0.07^b^	317.67 ± 5.201^a^	3.266 ± 0.025^a^
		*P* value	0.0002**	< 0.0001**	< 0.0001**	< 0.0001**	< 0.0001**
g.44601035G > A	1	AA(58)	6457.58 ± 264.57^c^	250.54 ± 10.739^b^	3.82 ± 0.106	200.21 ± 5.21^c^	3.136 ± 0.038
		AG(394)	6921.12 ± 251.62^a^	262.73 ± 10.226^a^	3.771 ± 0.101	211.76 ± 4.976^a^	3.113 ± 0.036
		GG(658)	6803.97 ± 250.4^b^	259.4 ± 10.176	3.795 ± 0.1	208.91 ± 4.949^b^	3.123 ± 0.036
		*P* value	< 0.0001**	0.0057**	0.2895	< 0.0001**	0.174
	2	AA(32)	10,135 ± 173.73^a^	359.67 ± 7.09	3.554 ± 0.07^b^	327.74 ± 5.172^a^	3.242 ± 0.025^a^
		AG(207)	9654.12 ± 133.79^c^	350.06 ± 5.63^b^	3.653 ± 0.054^b^	307.73 ± 4.103^b^	3.198 ± 0.018^a^
		GG(372)	9806.93 ± 131.91^b^	365.22 ± 5.56^a^	3.766 ± 0.054^a^	309.28 ± 4.053^b^	3.172 ± 0.018^b^
		*P* value	0.0004**	< 0.0001**	< 0.0001**	< 0.0001**	< 0.0001**
g.44,601,887 C > T	1	CC(468)	6862.52 ± 249.21^a^	252.95 ± 10.093^a^	3.722 ± 0.1^a^	210.89 ± 4.859^a^	3.141 ± 0.036^a^
		CT(521)	6954.1 ± 249.61^a^	252.42 ± 10.109^a^	3.662 ± 0.1^b^	212.4 ± 4.866^b^	3.123 ± 0.036^b^
		TT(138)	6655.56 ± 254.64^b^	240.09 ± 10.309^b^	3.66 ± 0.102	200.42 ± 4.958^b^	3.102 ± 0.037^b^
		*P* value	< 0.0001**	< 0.0001**	0.0028**	< 0.0001**	0.0003**
	2	CC(272)	9466.9 ± 132.53	366.55 ± 5.573^a^	3.904 ± 0.054^a^	302.28 ± 4.062^b^	3.197 ± 0.018
		CT(271)	9346.91 ± 130.18^a^	352.85 ± 5.484^b^	3.816 ± 0.053^b^	297.69 ± 3.997^c^	3.193 ± 0.018^b^
		TT(70)	9599.7 ± 150.37^b^	348.49 ± 6.221^b^	3.63 ± 0.061^c^	310.03 ± 4.536^a^	3.226 ± 0.021^a^
		*P* value	0.0113*	< 0.0001**	< 0.0001**	< 0.0001**	0.0441*
g.44602809G > T	1	GG(856)	6866.89 ± 252.49^a^	257.52 ± 10.247	3.741 ± 0.101	215.45 ± 4.972^a^	3.135 ± 0.036^b^
		GT(250)	6723 ± 255.19^b^	252.72 ± 10.362	3.76 ± 0.102	211.65 ± 5.035^b^	3.155 ± 0.037^a^
		TT(10)	6949.16 ± 328.85	258.12 ± 13.253	3.758 ± 0.131	220.38 ± 6.286	3.187 ± 0.048
		*P* value	0.0206*	0.0735	0.6532	0.0003**	0.0138*
	2	GG(464)	9540.27 ± 128.29^a^	359.12 ± 5.416^a^	3.78 ± 0.052	298.36 ± 3.947^a^	3.15 ± 0.018^a^
		GT(140)	9474.07 ± 136.04^a^	356.36 ± 5.688^a^	3.789 ± 0.055	293.69 ± 4.146^b^	3.12 ± 0.019^b^
		TT(9)	8906.48 ± 259.78^b^	332.56 ± 10.358^b^	3.773 ± 0.104	271.51 ± 7.559^c^	3.074 ± 0.038
		*P* value	0.0172*	0.0096**	0.9404	< 0.0001**	0.0016**
g.44603687G > A	1	AA(419)	6826.6 ± 249.82	247.54 ± 10.117^b^	3.66 ± 0.1^b^	208.66 ± 4.867^b^	3.118 ± 0.036^b^
		AG(536)	6899.47 ± 250.36	253.51 ± 10.138^a^	3.7 ± 0.1	209.67 ± 4.876	3.114 ± 0.036^b^
		GG(163)	6906.15 ± 251.7	253.89 ± 10.195	3.725 ± 0.101^a^	212.59 ± 4.912^a^	3.155 ± 0.036^a^
		*P* value	0.249	0.0035**	0.0226*	0.0084**	< 0.0001**
	2	AA(203)	9453.37 ± 132.73^b^	351.16 ± 5.568^b^	3.715 ± 0.054^a^	296.45 ± 4.059^b^	3.15 ± 0.018
		AG(306)	9450.28 ± 129.55^b^	351.05 ± 5.461^b^	3.742 ± 0.053^a^	293.46 ± 3.981^b^	3.13 ± 0.018
		GG(105)	9660.5 ± 142.9^a^	372.47 ± 5.954^a^	3.844 ± 0.058^b^	302.66 ± 4.34^a^	3.147 ± 0.02
		*P* value	0.0183*	< 0.0001**	0.0003**	0.0002**	0.073

Note: LSM ± SE: least squares mean ± standard deviation; the number in the bracket represents the number of cows for the corresponding genotype; *P* shows the significance level for the genetic effects of SNPs; Different superscript corresponding to the genotypes indicate significant differences between the genotypes; * indicates *P* < 0.05; ** indicates *P* < 0.01

In the first lactation, we observed all the 12 SNPs in *KLF6* showed significant associations with milk protein yield (*P* < 0.0001 ~ 0.0084), ten with milk fat yield (*P* < 0.0001 ~ 0.0383), nine with milk protein percent (*P* < 0.0001 ~ 0.0441), seven with milk fat percent (*P* < 0.0001 ~ 0.0335) and six with milk yield (*P* < 0.0001 ~ 0.0221). In the second lactation, all the 12 SNPs in *KLF6* were significantly associated with milk yield (*P* < 0.0001 ~ 0.0183) and protein yield (*P* < 0.0001 ~ 0.0221), ten with fat yield (*P* < 0.0001 ~ 0.096), nine with fat percent (*P* < 0.0001 ~ 0.0003) and seven with protein percent (*P* < 0.0001 ~ 0.0021).

In addition, as the results showed in Table S1, the additive, dominant, and substitution effects of the 12 SNPs on milk traits were significant as well (*P* < 0.05). The phenotypic variance ratios explained by these SNPs of five milk traits ranged from less than 0.0001–2.1301%, and the principal explanations were milk yield and protein yield (Table S2**)**.

### Associations between haplotype and the five milk traits

**Fig. 2:**
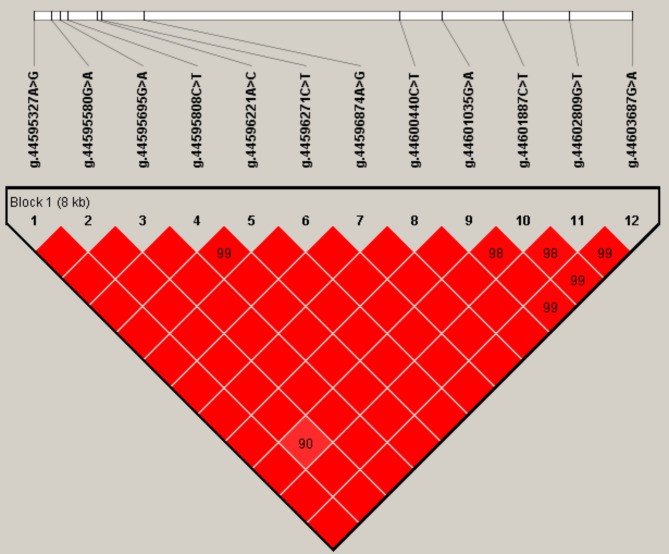
Linkage disequilibrium estimated among the 12 SNPs in *KLF6* (D′=0.829 ~ 1.0). The text above the horizontal numbers is the SNP names. The values within boxes are pairwise SNP correlations (D′), while bright red boxes without numbers indicate complete LD

We estimated the degree of linkage disequilibrium (LD) among the 12 SNPs in *KLF6* and inferred one haplotype block using Haploview4.2 (D′ = 0.829-1.0; Fig. 2). Regarding the block, the frequencies of the haplotype H1 (AGGCACACGCGG), H2 (GAACATGTATGA), H3 (GAATCCGCGCGA), H4 (GAATCCGCGCTA), H5 (GAACATGCGTGA) and H6 (GAACACGCGTGA) were 38.5%, 23%, 13.5%, 12.1%, 7.9% and 3.1%, respectively.

Similarly, the haplotype-based association analysis revealed that the haplotypes of *KLF6* were significantly associated with milk yield (*P* = < 0.0001), fat yield (*P* = 0.0203), protein yield (*P* = < 0.0001) and protein percent (*P* = 0.003) in the first lactation, and all the five milk traits in the second lactation (*P* = < 0.0001 ~ 0.009) (Table 3).

**Table 3:** Associations of haplotypes with milk production traits in two lactations in Chinese Holstein (LSM ± SE).

Lactation	Genotype (No.)	Milk yield, kg	Fat yield, kg	Fat percentage, %	Protein yield, kg	Protein percentage, %
1	H1H1(143)	8174.01 ± 97.282^a^	301.99 ± 4.1^a^	3.747 ± 0.039	256.71 ± 2.986^a^	3.163 ± 0.014^a^
	H1H2(184)	8270.28 ± 92.42^b^	302.13 ± 3.904^a^	3.683 ± 0.037	257.19 ± 2.843^a^	3.137 ± 0.013
	H1H3(108)	8114.28 ± 101.52^a^	301.1 ± 4.245	3.726 ± 0.041	251.54 ± 3.093^a^	3.113 ± 0.014^b^
	H2H2(55)	7648.66 ± 125.92^b^	288.37 ± 5.198^b^	3.765 ± 0.051	239.77 ± 3.789^b^	3.155 ± 0.018
	*P* value	< 0.0001**	0.0203*	0.1261	< 0.0001**	0.003**
2	H1H1(91)	9989.7 ± 116.78^a^	381.12 ± 4.945^a^	3.815 ± 0.047^a^	314.53 ± 3.603^aB^	3.153 ± 0.016^a^
	H1H2(103)	9683.65 ± 112.73^b^	356.15 ± 4.804^b^	3.704 ± 0.046^a^	305.36 ± 3.5^bC^	3.169 ± 0.016
	H1H3(55)	10,179 ± 130.48^a^	383.38 ± 5.486^a^	3.796 ± 0.053^a^	322.67 ± 3.998^AB^	3.19 ± 0.018
	H2H2(30)	10,259 ± 162.51^a^	362.23 ± 6.738^b^	3.502 ± 0.065^b^	330.86 ± 4.912^ A^	3.227 ± 0.023^b^
	*P* value	< 0.0001**	< 0.0001**	< 0.0001**	< 0.0001**	0.009**

Note: LSM ± SE: least squares mean ± standard deviation; the number in the bracket represents the number of cows for the corresponding genotype; *P* shows the significance level for the genetic effects of SNPs; Different superscript corresponding to the haplotypes indicate significant differences between the haplotypes; * indicates *P* < 0.05; ** indicates *P* < 0.01

### Functional variation prediction and verification caused by SNPs

We used Jaspar software to predict the transcription factor binding sites (TFBSs) change of the seven SNPs (g.44,595,327 A > G, g.44595580G > A, g.44595695G > A, g.44,595,808 C > T, g.44,596,221 A > C, g.44,596,271 C > T and g.44,596,874 A > G) in the 5′ flanking region of *KLF6*, and found that all the SNPs changed TFBSs (Table 4).

**Table 4:** Changes of TFBSs caused by the SNPs in the 5′flanking region of *KLF6.*

SNP	Allele	Transcription factor	Transcript relative score (≥ 0.85)	Predicted binding site sequence
g.44,595,327 A > G	A	SOX18	0.89	cagAatag
	G	GATA2	0.99	gGata
		GATA1	0.95	gGatag
		GATA3	0.91	gGatag
g.44595580G > A	G	EVX2	0.91	tctcattaaG
		EMX2	0.91	tctcattaaG
		EVX1	0.90	tctcattaaG
	A	NR1I3	0.91	ttaaActct
g.44595695G > A	G	FOXC1	0.86	atggtGta
	A	FOXL1	0.85	atggtAta
g.44,595,808 C > T	C	NPAS4	0.87	cttCgtgaggg
	T	SOX15	0.86	tcttTgtgag
g.44,596,221 A > C	A	KLF4	0.86	gAaggcaagg
	C	ATF1	0.91	gttacgCa
g.44,596,271 C > T	C	HIC2	0.91	gtgcCctgt
	T	SOX10	0.86	cTctgt
g.44,596,874 A > G	A	ERG	0.89	gggcAggaagggca
	G	KLF12	0.89	ggggcGgga

Note: the upper-case letters in the sequence are the SNP loci

**Fig. 3:**
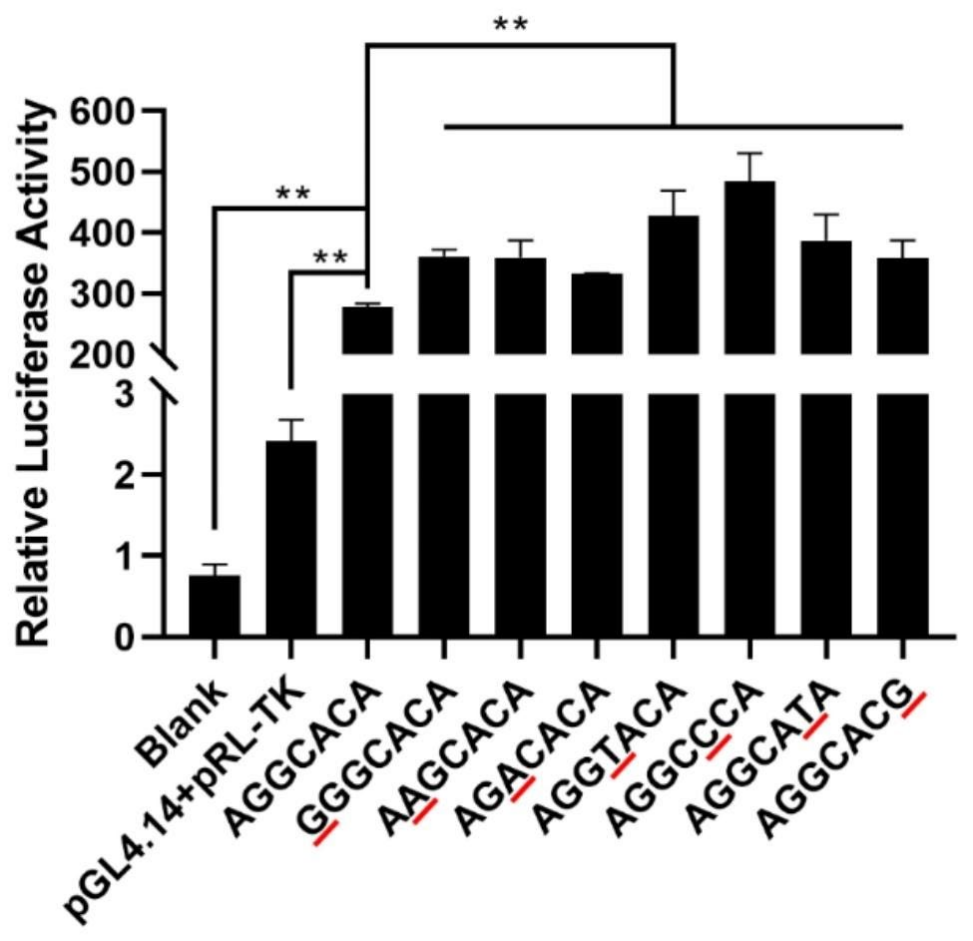
Luciferase assay result of the recombinant plasmids in HEK293 cells. Blank: blank cells. pGL4.14 + pRL-TK: empty vector. The nucleotides in red highlight referred to the mutation compared to the first plasmids. **: *P* < 0.01

Further, we performed the luciferase assay to validate the above prediction results (Fig. 3). The luciferase activities of the construct G of g.44,595,327 A > G, A of g.44595580G > A, A of g.44595695G > A, T of g.44,595,808 C > T, C of g.44,596,221 A > C, T of g.44,596,271 C > T, G of g.44,596,874 A > G were observed significantly higher than those of the blank control (*P* < 0.0001), empty vector pGL4.14 (*P* < 0.0001), and the construct A of g.44,595,327 A > G, G of g.44595580G > A, G of g.44595695G > A, C of g.44,595,808 C > T, A of g.44,596,221 A > C, C of g.44,596,271 C > T, A of g.44,596,874 A > G, respectively (*P* < 0.0001 ~ 0.0015). These results indicated the seven SNPs in the 5′ flanking region regulated the transcriptional activity of *KLF6* gene.

We utilized the RNAfold web server to predict the secondary structures of mRNA for the 5 SNPs in UTR and exon regions of *KLF6* gene (Table 5). When T replaced C in g.44,600,440 and A replaced G in g.44,603,687, the minimum free energy (MFE) of mRNA secondary structures decreased, and *KLF6* got more stable. Conversely, when T replaced C in g.44,601,887 and G in g.44,602,809, the MFE of mRNA secondary structures increased, and *KLF6* got more unstable. These observations suggested that the 4 SNPs might change the *KLF6* mRNA secondary structure to affect the *KLF6* expression.

**Table 5:** The minimum free energy (MFE) values of optimal secondary structure of *KLF6* mRNA.

SNP	Allele	MFE (kcal/mol)
g.44,600,440 C > T	C	-1982.3
	T	-1983.3
g.44601035G > A	G	-1982.3
	A	-1982.3
g.44,601,887 C > T	C	-1982.3
	T	-1980.1
g.44602809G > T	G	-1982.3
	T	-1980.7
g.44603687G > A	G	-1982.3
	A	-1983.0

Note: MFE: minimum free energy

Furthermore, we employed the SOPMA software to predict the protein secondary structure for the missense mutation (g.44601035G > A) in exon 2 of the *KLF6* gene. The analysis revealed that the beta turn was changed from 6.47 to 5.83% and the random coil from 60.84 to 61.49%. However, according to the prediction result from PROVEAN, there was no observed change in the protein function (score = -0.038).

## Discussion

Our previous liver transcriptome study of Holstein cows identified *KLF6* gene as a promising candidate affecting milk production traits. Not only did it exhibit differential expression across various lactation periods, but it was also found to be intricately linked to the PPARα signaling pathway and located near the known QTLs for milk yield and composition traits. In this study, through single locus and haplotype-based association analysis, we confirmed the significant genetic effects of *KLF6* gene on milk yield, milk fat and protein traits in dairy cattle.

The *KLF6* gene was highly expressed in various tissues and played a critical role in adipocyte differentiation by activating PPARα that regulated hepatic steatosis, lipoprotein synthesis, hepatic gluconeogenesis and fatty acid transport proteins in mouse [31–33]. *KLF6* also promoted preadipocyte differentiation by suppressing a factor named delta-like1 (DLK1), which acted by maintaining the preadipocyte state and preventing adipocyte differentiation in mouse preadipocytes [34, 35]. These studies demonstrated the involvement of *KLF6* gene in lipid metabolism, and were consistent with the results in this study that *KLF6* gene has significant effects on milk fat traits. In addition, we found *KLF6* gene was significantly associated with milk protein traits as well. This is probably due to the high genetic correlation between milk fat and protein traits in dairy cattle [36–38].

Transcription factors, which belong to the family of sequence-specific DNA-binding proteins, regulate gene expression across diverse organisms [39, 40]. Specifically, SNPs located within transcription factor binding sites (TFBSs) can contribute to the allele-specific binding of transcription factors. In the present study, we identified 7 SNPs within 5’ regulatory region of the *KLF6* gene that altered TFBSs, subsequently activating the *KLF* expression. These TFBS modifications corresponded to transcription factors GATA2, GATA1, GATA3, NR1I3, FOXL1, SOX15, ATF1, SOX10 and KLF12 which have been shown to promote the expression of target genes in human and mouse in several prior studies [41–48]. Conversely, transcription factors SOX18, EVX2, EMX2, EVX1, FOXC1, NPAS4, KLF4, HIC2 and ERG have been reported to inhibit the expression of their target genes [49–56]. In this study, for the g.44,595,808 C > T and g.44,596,221 A > C, the positive genetic effects of alleles T and C on milk protein may be due to the activated *KLF6* expression induced by transcription factors NR1I3 and ATF1, respectively. The regulatory roles of these SNPs on *KLF6* expression thereby impacting milk traits need more in-depth investigations to be validate.

Furthermore, in our study, the missense mutation in exon 2, g.44601035G > A, was predicted to impact the secondary structure of the KLF6 protein. As the backbone of advanced RNA function, the secondary structure of mRNA plays a pivotal role in various biological processes, including protein folding and transport, initiation and extension of translation process, regulation of translation rate, and directly affects the stability of mRNA itself [57, 58]. In addition, the secondary structures of *KLF6* mRNA corresponding to allele T of g.44,600,440 C > T, allele C of g.44,601,887 C > T, allele G of g.44602809G > T and allele A of g.44603687G > A exhibited increased stability in comparison to the alleles C, T, T and G, respectively. The enhanced stability of the mRNA secondary structure associated with allele C of g.44,601,887 C > T may provide insight into its active genetic effects on milk yield, fat yield, and protein traits.

Studies have shown that the incorporation of functional gene information associated with substantial genetic effects on target breeding traits can improve the accuracy of genomic evaluation [59, 60]. These significant SNPs identified in this study could be used as molecular markers for genetic improvement programs of dairy cattle through genomic selection.

## Conclusion

Based on our preceding RNA-seq investigation that identified *KLF6* genes as a promising candidate for milk traits in dairy cattle, this study first demonstrated the significant genetic effects of *KLF6* gene on milk yield and composition traits. A total of 12 SNPs were identified, 7 of them altered the transcriptional activity of *KLF6*, 4 changed the *KLF6* mRNA secondary structures, and 1 missense mutation changed KLF6 protein secondary structure. These SNPs could be used as valuable genetic markers for molecular breeding program by incorporating them into genomic selection chips in dairy cattle.

## Materials and methods

### Animals and phenotypic data

We gathered the blood samples of 1123 Chinese Holstein cows from 80 sire families from Qingyuanlvze and Shuangfeng dairy farms (Baoding, Hebei, China) in Hebei province. The pedigree and phenotypic data of five milk traits, containing milk yield, milk fat yield, milk fat percentage, milk protein yield, and milk protein percentage were provided by the Hebei Province Animal Husbandry and Fine Breeds Work Station (Shijiazhuang, Hebei, China). Descriptive statistics of the phenotypic values for milk traits in the first and second lactations were presented in Table 6.

**Table 6:** Descriptive statistics of the phenotypic values for milk production traits

	Mean		Standard deviation		Maximum		Minimum		Coefficient of variation	
Lactation	1	2	1	2	1	2	1	2	1	2
Milk yield, kg	8140.13	9735.07	1467.72	1750.93	12536.44	15016.28	3721.48	4335.76	0.18	0.18
Fat yield, kg	304.08	367.27	72.29	97.83	562.43	676.50	98.57	153.94	0.24	0.27
Fat percentage, %	3.77	3.79	0.76	0.84	6.97	6.61	2.00	2.01	0.20	0.22
Protein yield, kg	257.06	309.70	46.03	55.87	398.19	466.83	114.16	154.87	0.18	0.18
Protein percentage, %	3.17	3.20	0.26	0.29	4.01	4.06	2.34	2.33	0.08	0.09

### SNP identification and genotyping

We extracted genomic DNA from whole blood samples of 1123 cows using the TIANamp Blood DNA Kit (Tiangen, Beijing, China) and measured the quantity and quality of DNA samples with NanoDrop 2000 spectrophotometer (Thermo Scientific, Hudson, NH, USA) and agarose gel electrophoresis (1.5%), respectively. Eighteen pairs of primers were designed with Primer3web version 4.1.0 (https://bioinfo.ut.ee/primer3/) and synthesized by the Beijing Genomics Institute (Beijing, China) to amplify the entire coding sequence and 2000 bp of the 5’ and 3’ flanking regions for the bovine *KLF6* gene in accordance with its genomic sequence (GenBank IDs: NC 037340.1) (Table S3).

We randomly selected 111 blood DNA samples from the 80 sire families to construct five DNA pools at the same concentration of 50 ng/µL, including one DNA pool with 23 samples and four pools with 22 samples in each. Based on the DNA pools as templates for PCR amplification (Table S4), the PCR products were bi-directionally sequenced on ABI3730XL DNA analyser (Applied Biosystems, Foster City, CA, USA) and aligned to the bovine reference sequences (ARS-UCD1.2) with BLAST (https://blast.ncbi.nlm.nih.gov/Blast.cgi) to identify potential SNPs. The matrix-assisted laser desorption/ionization-time of flight mass spectrometry assay (MALDI-TOF MS, Sequenom MassARRAY, Agena, San Diego, USA) was performed for subsequent individual genotyping of the identified SNPs for the 1123 Holstein cows.

### Linkage disequilibrium (LD) estimation

We calculated the extent of LD among the 12 identified SNPs in *KLF6* with Haploview 4.2 (Broad Institute, Cambridge, MA, USA). The D’ or r^2^ value was used to measure the degree of LD. The Haplotypes with frequencies of less than 0.05 were discarded.

### Association analyses between SNPs/ haplotypes on milk production traits

The pedigrees of 1123 individuals were traced back three generations for the association studies between the detected SNPs or haplotypes and five milk traits. SAS 9.2 mixed procedure was performed with the following model: Y = µ + hys + b × M + G + a + e, where Y is the phenotypic value of five milk traits of each cow; µ is the overall mean; hys is the fixed effect of farm (1–2 for the two farms, respectively), calving year (1–7 for the years of 2013–2019, respectively), and calving season (1 for April to May; 2 for June to August; 3 for September to November, and 4 for December to March); b is the regression coefficient of covariant M; M is calving age as a covariant; G is the genotype or haplotype combination effect; a is the individual random additive genetic effect, distributed as ${\text{N}}(0,{\text{A}}\delta _{\text{a}}^2)$, with the additive genetic variance ${{\delta }}_{\text{a}}^{2}$; and e is the random residual effect, distributed as $\text{N} \left(0, \text{I}{{\delta }}_{\text{e}}^{2}\right)$, with identity matrix I and residual error variance ${{\delta }}_{\text{e}}^{2}$. Multiple tests were implemented by Bonferroni correction, with the significance level equal to the original *P* value divided by the number of genotype or haplotype combinations.

In addition, the additive (a), dominant (d), and substitution (α) effects were also computed as follows: $$\text{a}=\frac{\text{A}\text{A}-\text{B}\text{B}}{\begin{array}{c}2\\ \end{array}}$$,$$\text{d}=\text{A}\text{B}-\frac{\text{A}\text{A}+\text{B}\text{B}}{\begin{array}{c}2\\ \end{array}}$$,${\alpha }=\text{a}+\text{d}(\text{q}-\text{p})$, where AA, AB, and BB were represented the least square means of milk traits attributes matching to genotypes, while p was the allele frequency of A and q was the allele frequency of B.

The phenotypic variance ratio of five milk traits explained by SNPs was calculated as follow: phenotypic variance ratio = $\,{{2pq{\alpha ^2}} \mathord{\left/{\vphantom {{2pq{\alpha ^2}} {\sigma _p^2}}} \right.\kern-\nulldelimiterspace} {\sigma _p^2}}$, where p and q were the allele frequencies of A and B, α was substitution effects and ${\sigma }_{p}^{2}$ was the phenotypic variance of the target trait.

### Biological function prediction

Using the Jaspar software (http://jaspar.genereg.net/) to determine whether SNPs in 5′ flanking region of the *KLF6* gene altered transcription factor binding sites (TFBSs) (relative score ≥ 0.85).

To predict changes in mRNA secondary structures for SNPs in UTR and exon regions, RNAfold Web Server (http://rna.tbi.univie.ac.at/cgi-bin/RNAWebSuite/RNAfold.cgi) was used. The minimum free energy (MFE) of the optimal secondary structure reflects the stability of mRNA structure. A lower MFE value indicates greater stability in the mRNA structure.

To predict the changes in protein secondary structure caused by missense mutation in the coding regions of genes, we applied SOPMA (https://npsa-prabi.ibcp.fr/cgi-bin/npsa automat.pl?page=/NPSA/npsa sopma.html) with the following parameters: similarity threshold: 8; number of states: 4-Helix, sheet, Turn, Coil; output width: 70.

Subsequently, we implemented PROVEAN software (http://sift.jcvi.org/index.php) to confirm whether the missense mutation changed protein function. The threshold value for PROVEAN is set at -2.5, below which alterations in protein function are considered.

### Construction of recombinant plasmid, cell culture and luciferase assay

**Fig. 4:**
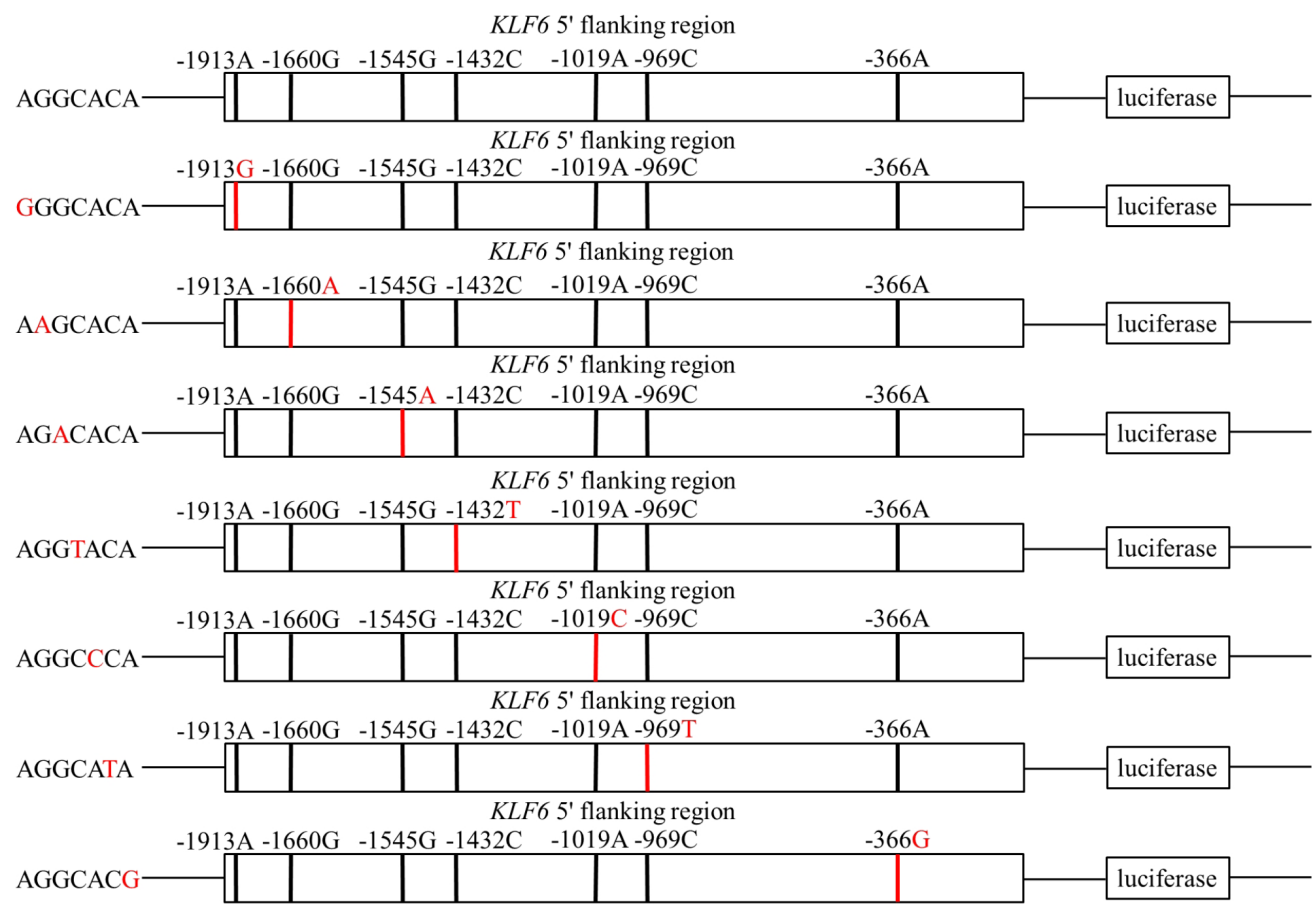
Sketches of recombinant plasmids. The nucleotides in red highlight referred to the mutation compared to the first plasmids

To examine the effects of the SNPs that were predicted to alter the transcription factor binding sites in the 5′ flanking region of *KLF6*, a total of 8 luciferase reporter gene fragments (Fig. 4) were designed and synthesized in Hitrobio (Beijing, China). These fragments contained 2000 bp of the 5′ flanking sequences of KLF6, with XhoI and HindIII restriction sites at the 5′ and 3′ termini, and were cloned into the pGL4.14 Luciferase Assay Vector (Promega, Madison, USA). These plasmids were subsequently purified using the EndoFree Mini Plasmid Kit II (Tiangen, Beijing, China), and their integrity of each construct’s insertions were sequenced to verify.

Human Embryonic Kidney (HEK)-293 T cells (Procell, Wuhan, China) were cultured at 37 °C and 5% CO_2_ with Dulbecco’s modified Eagle’s medium (DMEM) supplemented with 10% heat-inactivated fetal bovine serum (Biosun, Shanghai, China). Approximately 1 × 10^5^ cells were seeded into 48-well plates and co-transfected using Lipofectamine 3000 (Invitrogen, CA, USA) with 200 ng of the constructed plasmid DNA and 20 ng of pRL-TK renilla luciferase reporter vector (Promega, WI, USA) per well. Three replicates were conducted for each construct. After 36 h, the 293T cells were collected and then the activities of firefly and renilla luciferases were measured using the DualLuciferase Reporter Assay System (Promega, WI, USA) with a multifunctional microplate detection system (BioTek, NY, USA). The average of three replicates was then calculated to obtain the normalized luciferase data (firefly/renilla).

### Electronic supplementary material

Below is the link to the electronic supplementary material.

Supplementary Material 1

## Data Availability

The information of genetic polymorphisms loci can be inquired on European Variation Archive (EVA; https://www.ebi.ac.uk/eva/) using the RS IDs (rs41692335, rs110464810, rs110289079, rs208019372, rs211266130, rs29024529, rs41692337, rs209109676, rs211273884, rs41692344, rs208700974 and rs41692345) in Table 2. The datasets generated during and analyzed during the current study are available in the article and its additional files.

## Abbreviations

a: Additive effect
CDS: Coding sequence
C/EBPα: CCAAT enhancer-binding proteins alpha
C/EBPβ/γ: CCAAT enhancer-binding proteins beta/gamma
d: Dominant effect
*DLK1*: Delta-like 1
*KLF6*: Kruppel Like Factor 6
LD: Linkage disequilibrium
MFE: Minimum free energy
PCR: Polymerase chain reaction
PPARα: Peroxisome proliferator-activated receptor alpha
PPARγ: Peroxisome proliferator-activated receptor-gamma
PPARγ2: Peroxisome proliferator-activated receptor-gamma 2
QTL: Quantitative trait loci
SNP: Single nucleotide polymorphism
SP/KLF: Specificity proteins/Krüppel-like factor
SREBP1: Sterol regulatory element-binding protein 1
TF: Transcription factor
TFBS: Transcription factor binding site
UTR: Untranslated region
α: Substitution effect
